# Estimating the Prevalence of Generative AI Use in Medical School Application Essays: Cross-Sectional Study

**DOI:** 10.2196/96673

**Published:** 2026-09-03

**Authors:** Nicholas C Spies, Valerie S Ratts, Ian S Hagemann

**Affiliations:** 1 Department of Pathology University of Utah Salt Lake City, UT United States; 2 Department of Obstetrics and Gynecology School of Medicine Washington University in St. Louis St. Louis, MO United States; 3 Department of Pathology and Immunology School of Medicine Washington University in St. Louis St. Louis, MO United States

**Keywords:** medical schools, artificial intelligence, AI, medical students, student selection

## Abstract

**Background:**

Generative AI tools became widely available to the public in November 2022. The extent to which these tools have been used by medical school applicants during the admissions process is unknown.

**Objective:**

We aimed to estimate the extent of generative AI use among cohorts of applicants spanning the rollout of these tools.

**Methods:**

We retrospectively analyzed 6000 essays from 2364 applicants submitted to a US medical school in 2021 to 2022 (baseline, before the wide availability of AI) and 2023 to 2024 (test year) to estimate the prevalence of AI use and its relation to other application data. We used GPTZero, a commercially available detection tool, to generate a metric (P_human_) reflecting the predicted probability that each essay was completely human generated, ranging from 0 (the essay appears to be entirely AI generated) to 1 (the essay appears to be entirely human generated).

**Results:**

Fully human-generated negative controls demonstrated a median P_human_ of 0.93 (range 0.89-0.97), while fully AI-generated positive controls demonstrated a median P_human_ of 0.01 (range 0.00-0.01). The “Personal Comments” essays submitted in the 2023 to 2024 application cycle had a median P_human_ of 0.77 (95% CI 0.76-0.78) compared with 0.83 (95% CI 0.82-0.85) during the 2021 to 2022 cycle. Approximately 12.3% and 2.7% of essays were evaluated as having P_human_ <0.5 in the test and baseline years, respectively. Essays submitted as part of the secondary application demonstrated lower P_human_ values than those of the American Medical College Application Service (AMCAS) “Personal Comments” essays. In applicant-clustered, multivariable generalized estimating equation analyses, supplementary essay type and younger age were significantly associated with lower P_human_. Application completion date, self-reported gender, program type (MD vs MD-PhD), grade point average (GPA), Medical College Admission Test (MCAT) score, socioeconomic status, and undergraduate major were not significant predictors after false discovery rate correction. P_human_ was not predictive of interview invitation or acceptance in adjusted applicant-level logistic regression analyses.

**Conclusions:**

An AI detection algorithm identified signs of increased use of generative AI in 2023 to 2024 medical school admission applications compared to those in the 2021 to 2022 baseline period, before AI was widely available. AI use did not appear to confer an admissions advantage. Although these results provide information about the applicant pool as a whole, AI detection is imperfect. We do not recommend deploying AI detection for individual applications in live admissions cycles.

## Introduction

Generative AI has exploded in popularity and availability in recent years, with “chatbots” driven by large language models (LLMs) providing a means to rapidly accomplish text-oriented tasks. These tools have a myriad of potential applications in medical education, including information retrieval [1] and the generation of practice questions [2], clinical vignettes, and simulations [3]. However, they also threaten the pedagogical value of writing assignments by allowing students to produce responses with minimal effort or understanding [4]. AI’s role in medical education remains a rapidly evolving field.

One setting where AI chatbots may have far-reaching implications is the admissions process. Medical school application essays are intended to give admissions officers information about applicants’ interests, experiences, attributes, and motivations. Moreover, the essays can approximate the work quality that can be expected of students if they ultimately matriculate. In November 2022, the first widely available chatbot, ChatGPT 3.5, was released to the public, introducing the possibility that medical school applicants could use this tool either to write their essays outright or for subtasks such as drafting or editing.

There is no consensus on the extent to which AI use by medical applicants is acceptable. Applicants have long relied on aids such as spelling and grammar checkers, formal editing services, and feedback from friends, family, and advisers. Receiving help from a chatbot could level the playing field for applicants with less access to conventional writing aids. On the other hand, AI-generated essays provide less insight into the applicant’s authentic self and work performance and are therefore less fit for purpose as components of a school application. For the 2023 to 2024 application cycle, the American Medical College Application Service (AMCAS) adopted a policy stating that essays must “not be written, in part or in whole, by another author and...not [be] the product of artificial intelligence” [5]. Although all applicants attested to this certification statement, it is uncertain to what degree they complied.

The popularity, potential impact, and ethical considerations of AI-driven chatbots have motivated the development of methods to distinguish AI-generated text from human-generated text. One reported method relies on the extent to which each word is predictable based on those that came before it (“perplexity”) and the degree of variation in sentence length and structure (“burstiness”) [6]. Human-generated writing tends to have higher perplexity and burstiness than the output of current LLMs, reflecting the greater variety and spontaneity of natural human expression. In this model, AI use is not detected directly; instead, detection of an AI-related style signal is used as a surrogate, with caveats.

An effective AI detector must be both sensitive and specific, similar to other diagnostic tests. In a benchmarking study, GPTZero attained an area under the receiver operating characteristic (AUROC) curve of greater than 96% on diverse writing samples, including product reviews, blogs, news stories, fiction, and, perhaps most relevant to this study, résumés written by a variety of LLMs [7]. We thus considered it fit for purpose for our study.

To explore the extent to which LLMs may be used in medical school applications, we conducted a retrospective study of AMCAS writing samples from before and after the surge in popularity of AI-driven chatbots in 2022. We analyzed these essays using GPTZero for AI detection [6] and compared the detector’s outputs across relevant demographic and essay-related metadata.

## Methods

### Data Retrieval

We conducted this study at a US medical school that participates in the AMCAS Data Exchange Service. Applicants were included in the study if they selected the school as part of their AMCAS application, regardless of whether they completed a secondary application. We retrieved data from the school’s applicant tracking system, identified only by a coded identifier. An honest broker who was not a member of the study team provided the data to eliminate the possibility of reidentification. We coded an application as “complete” if the student submitted an AMCAS file, letters of recommendation meeting the school’s requirement, and a secondary application. We retrieved the following data from the AMCAS application: age, self-reported gender, program type (MD vs MD-PhD), visa status, socioeconomic status indicator, undergraduate majors, undergraduate grade point average (GPA), highest Medical College Admission Test (MCAT) 3-digit score, and the applicant’s personal statement. We coded socioeconomic status using the AMCAS indicator EO1 and/or EO2 (indicating that neither parent has a college degree or holds an executive, managerial, or professional occupation) vs other. We manually coded each applicant’s undergraduate majors as follows: science, technology, engineering, and mathematics (STEM); non-STEM; or both (applicable only to applicants declaring multiple majors; Table S1 in Multimedia Appendix 1). Visa status was retrieved as an imperfect surrogate for native English proficiency. We retrieved the following data from the school-specific secondary application: a supplementary essay in which the student was asked to describe a time in life when they were unsuccessful (“Failure” essay, optional in 2021-2022 but required in 2023-2024), a free-text box where they could enter any additional information they wished to share (“Anything Else” essay, optional), and the date of application submission. Some applicants lacked a submission date, indicating that they failed to assemble a complete file (usually due to not submitting their secondary application). Each student’s interview decision (interview vs no interview) and admission decision (accepted vs rejected) were extracted from our database. We considered placement on the Alternate List as rejection for this study.

Within each application cycle, we constructed separate extracts for the “Personal Comments,” “Failure,” and “Anything Else” essays. A random sample of 1000 “Personal Comments” essays from each year was extracted. Then, any “Failure” or “Anything Else” essay for those applicant IDs was specifically included. Given the optional nature of the secondary essays and the fact that some students did not submit their secondary applications, the extraction set was supplemented with another random sampling of secondary essays to complete the remaining cohort to 1000 essays of each type. This sampling approach enabled us to perform within- and across-applicant analyses to better characterize the association among year, applicant, and essay type with the likelihood of AI use.

### Detecting Evidence of AI Use in Applicant Essays

We used the GPTZero API (version 2.0.0; Superhuman), a commercially available web-based service, to detect AI use. We sent the entirety of each writing sample to the API for analysis, which returned a vector (class_probabilities) of 3 elements: the model’s estimated probabilities that the document was fully generated by a human, fully generated by AI, or mixed [8]. For downstream analysis, we used the first of these outputs, denoted as P_human_. This measure is not the proportion of the essay predicted to be human-written, although there is likely to be a correlation. In essays written with heavy use of AI, many sentences will be flagged as AI-written or mixed; these sentences will have a low detected probability of being human-written, and the overall P_human_ will be low. Essays with mainly human-written sentences will, conversely, have a high sentence-wise and document-wise predicted probability of being written by a human and, therefore, a high P_human_. A P_human_ of <0.5 denotes an input that was more likely than not written with the help of AI.

Given the reported difficulties in accurately discriminating between human and AI-generated text [9], we first performed a preliminary analysis using negative and positive controls. We defined continuation criteria for the real-world analysis as the detection of a difference in predicted probability of greater than 0.5 with 95% confidence (eg, a median human probability of >0.75 in negative controls and <0.25 in positive controls). For negative controls, we subjected 5 essays known to be completely human generated, obtained from the authors’ personal files, to the workflow described below. For positive controls, we submitted essay prompts to GPT 3.5 (OpenAI) and Claude 3 Sonnet (Anthropic Inc) through their respective online user interfaces. The 52 responses from each chatbot (104 total) were completely generated by the LLM, with no subsequent human editing. The prompts and resulting positive-control essays are available in Table S2 in Multimedia Appendix 2.

### Statistical Analysis

We performed all analyses in R (version 4.5.1; R Foundation for Statistical Computing) using the *tidyverse* [10] framework and generated tables using *gtsummary* [11]. The code is available at [12].

We used Gaussian linear models for univariate analyses of application factors. To account for repeated essays in the application cycle analysis, we fit a Gaussian identity-link generalized estimating equation (GEE) containing application cycle, essay type, and their interaction, with robust sandwich SEs clustered by coded AMCAS identifier. The independence working correlation retained applicants represented by 1, 2, or 3 essays, and overall application cycle means were standardized with equal weight across the 3 essay types. We also fit an exchangeable-working-correlation GEE as a sensitivity analysis.

We also conducted 2 additional clustered multivariable sensitivity analyses of the 2023 to 2024 essays: one restricted to applicants classified as having a completed application using the application completion indicator (a nonmissing application submission date), and one restricted to applicants who submitted all 3 essay types. These models used the same GEE specification and covariates as the primary model, except that application completion was omitted because it was fixed in the completed application subset and nearly invariant in the all-3-essay subset. Full specifications are provided in Multimedia Appendix 3.

### Ethical Considerations

The Human Research Protection Office at our institution determined that this study did not constitute human subjects research (202401024, issued on January 16, 2024). Moreover, permission was obtained from the Association of American Medical Colleges to use AMCAS data for this study. Privacy and confidentiality were maintained by labeling data only with a coded identifier; an honest broker who was not a study author held the key and removed other personally identifiable information. There was no compensation to participants.

## Results

### Selection of Essays for Analysis

In the 2021 to 2022 application cycle, the school received 6137 applications before the November 15, 2021, deadline; in the 2023 to 2024 application cycle, it received 5055 applications before the November 15, 2023, deadline. We analyzed 1000 selected “Personal Comments,” 1000 “Failure,” and 1000 “Anything Else” essays from each cycle, for a total of 3000 essays per cycle and 6000 overall. The selected essays represented 1184 applicants in the 2021 to 2022 application cycle and 1189 in the 2023 to 2024 application cycle (Table 1). Applicants contributed a mean of 2.53 (SD 0.75) and 2.52 (SD 0.76) selected essays, respectively; 819 (69.2%) and 818 (68.8%) contributed all 3 essay types.

**Table 1:** Characteristics of applicants represented in the essay sample.

Characteristics			2021 to 2022 (n=1184)		2023 to 2024 (n=1189)		*P* value^a^	
Age (years), median (IQR)			22.6 (21.6-23.7)		22.7 (21.8-23.9)		*.03*	
Self-reported gender, n (%)								*.002*
	Female	632 (53)		592 (50)				
	Male	550 (46)		582 (49)				
	Other	2 (0.2)		15 (1.3)				
Program, n (%)								.17
	Regular MD	1057 (89)		1040 (87)				
	Combined MD-PhD	127 (11)		149 (13)				
Visa status, n (%)								.83
	US citizen	1100 (93)		1102 (93)				
	Other visa status	84 (7.1)		87 (7.3)				
American Medical College Application Service socioeconomic status, n (%)								.58
	EO1 and/or EO2	159 (13)		169 (14)				
	Other	1025 (87)		1020 (86)				
Grade point average, median (IQR)			3.89 (3.72-3.97)		3.91 (3.77-3.98)		*<.001*	
Medical College Admission Test, median (IQR)			518 (514-521)		518 (514-521)		.96	
Completed application, n (%)			1098 (93)		1105 (93)		.85	
Invited to interview, n (%)			300 (25)		297 (25)		.84	
Accepted, n (%)			100 (8.4)		93 (7.8)		.58	
Essays included per student, mean (SD)			2.53 (0.75)		2.52 (0.76)		.78	

^a^*P* values were calculated using the Wilcoxon rank-sum test or the Pearson chi-square test, as appropriate. Italics indicate *P*≤.05.

We hypothesized that AI use would be rare or absent in the baseline year (2021-2022), leading the detector to produce P_human_ probabilities near 1.00. Conversely, in the first application cycle after the widespread availability of generative AI tools (2023-2024), we expected a proportion of students to have written some or all of their essays with these tools, leading to lower P_human_ estimates.

### Labeling of Human-Generated Negative Controls and AI-Generated Positive Controls by GPTZero

A series of negative controls known to be completely human generated demonstrated a median P_human_ of 0.93 (95% CI 0.86-1), with a representative essay yielding human, AI, and mixed scores of 0.945, 0.054, and 0.001, respectively. Conversely, a series of completely AI-generated positive controls, for which no downstream editing was performed, demonstrated a median P_human_ of 0.01 (95% CI 0-0.05), with a representative essay yielding human, AI, and mixed scores of 0.001, 0.997, and 0.002, respectively. These results met our continuation criteria, and we proceeded with our planned study.

### Comparison of Estimated Human-Generated Probabilities Between the 2023-2024 and 2021-2022 Application Cycles

**Figure 1:**
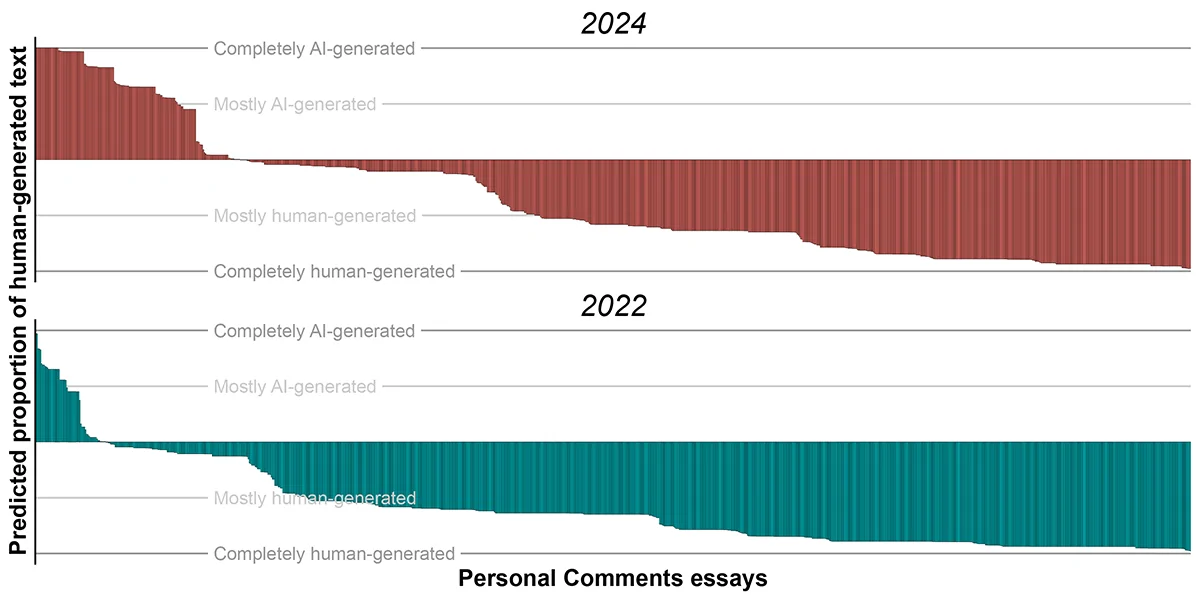
Waterfall plot of GPTZero-estimated probabilities that 1000 selected “Personal Comments” essays were AI or human generated. The 2024 application cycle (red, top) is compared with the pre-ChatGPT baseline 2022 cycle (tan, bottom).

We compared P_human_ values for 1000 selected “Personal Comments” essays from the 2023 to 2024 application cycle to those from the pre-ChatGPT 2021 to 2022 application cycle (Figure 1). The median P_human_ was 0.77 in the 2023 to 2024 cycle (95% CI 0.76-0.78) compared to 0.83 in the 2021 to 2022 baseline (95% CI 0.82-0.85). Overall, 12.3% of essays had P_human_<0.5 in the 2023 to 2024 cycle. In contrast, only 2.7% of essays had P_human_<0.5 in the baseline cycle, significantly fewer than that in the first ChatGPT year (Fisher exact test: *P*<.001).

Across all 3 essay types, the applicant-clustered GEE estimated a difference in P_human_ of −14.5% between the 2023 to 2024 and 2021 to 2022 application cycles, standardized with equal weight across essay types (robust SE 0.0099, 95% CI −16.4% to −12.5%; *P*<.001). In other words, after accounting for the correlation among essays from the same applicant, the probability that an essay was classified as fully human generated was 14.5 percentage points lower in the 2023 to 2024 cohort than in the pre-ChatGPT baseline.

### Analysis of Factors Associated With GPTZero-Estimated Human-Generated Probabilities

We analyzed the 1000 selected “Personal Comments,” “Failure,” and “Anything Else” essays from the 2023 to 2024 cycle using GPTZero’s AI detection algorithm. These 3000 essays represented 1189 unique applicants, as some applicants did not contribute every secondary essay type. We used the predicted probability that each essay was entirely human generated (P_human_) as the outcome for univariate linear models and the primary applicant-clustered multivariable GEE. Predictors were essay type, application completion, age, self-reported gender, program type, visa status, socioeconomic status, GPA, and MCAT score.

In the univariate analysis (Table 2), the essays in the secondary application had lower P_human_ than the universal “Personal Comments” essay by an average of 5% to 9% (*P*<.001). For every 1-year increase in applicant age, there was an average of a 1% increase in P_human_ (*P*<.001). Essays submitted by applicants requiring a visa to study in the United States had P_human_ values that were 8% lower than those submitted by US citizens (*P*<.001). For each 0.1-point increase in GPA, P_human_ was 1% lower (*P*<.001). Incomplete applications had P_human_ values that were 9% higher than those of complete applications (*P*=.01). No significant differences were observed based on program type, self-reported gender, socioeconomic status, or MCAT score.

**Table 2:** Univariate analysis of application-related factors as predictors of P_human_, the GPTZero-predicted probability that a document was fully human generated (N=3000).

Characteristics			Essays, n		Effect size^a^		95% CI		*P* value		q value^b^
Essay type											
	Personal Comments	1000		—^c^		—		—		—	
	Failure	1000		0.91		0.88-0.93		<.001		*<.001*	
	Anything Else	1000		0.95		0.92-0.97		<.001		*<.001*	
Age (years)			3000		1.01		1.01-1.01		<.001		*<.001*
Self-reported gender											
	Female	1494		—		—		—		—	
	Male	1474		1.00		0.98-1.03		.73		.73	
	Other	32		1.11		0.99-1.24		.08		.11	
Program											
	Regular MD	2630		—		—		—		—	
	Combined MD-PhD	370		1.02		0.98-1.05		.40		.48	
Visa status											
	US citizen	2771		—		—		—		—	
	Other visa status	229		0.92		0.88-0.96		<.001		*<.001*	
Medical College Admission Test			2998		1.00		1.00-1.00		.04		.06
Grade point average			3000		0.99		0.98-1.00		<.001		*<.001*
Application completion											
	Completed	2911		—		—		—		—	
	Not completed	89		1.08		1.02-1.14		.01		*.03*	

^a^Effect=e^β^ in the regression model, representing the average fold change in estimated P_human_ associated with a unit increment in each feature. For age, effect size gives the increase associated with a unit increase of 1 year; for Medical College Admission Test, a 1-point increase in 3-digit score; and for GPA, a 0.1-point increase in undergraduate GPA.

^b^False discovery rate–adjusted *P* values for multiple testing. Italics indicate *q*≤0.05.

^c^Not applicable.

**Figure 2:**
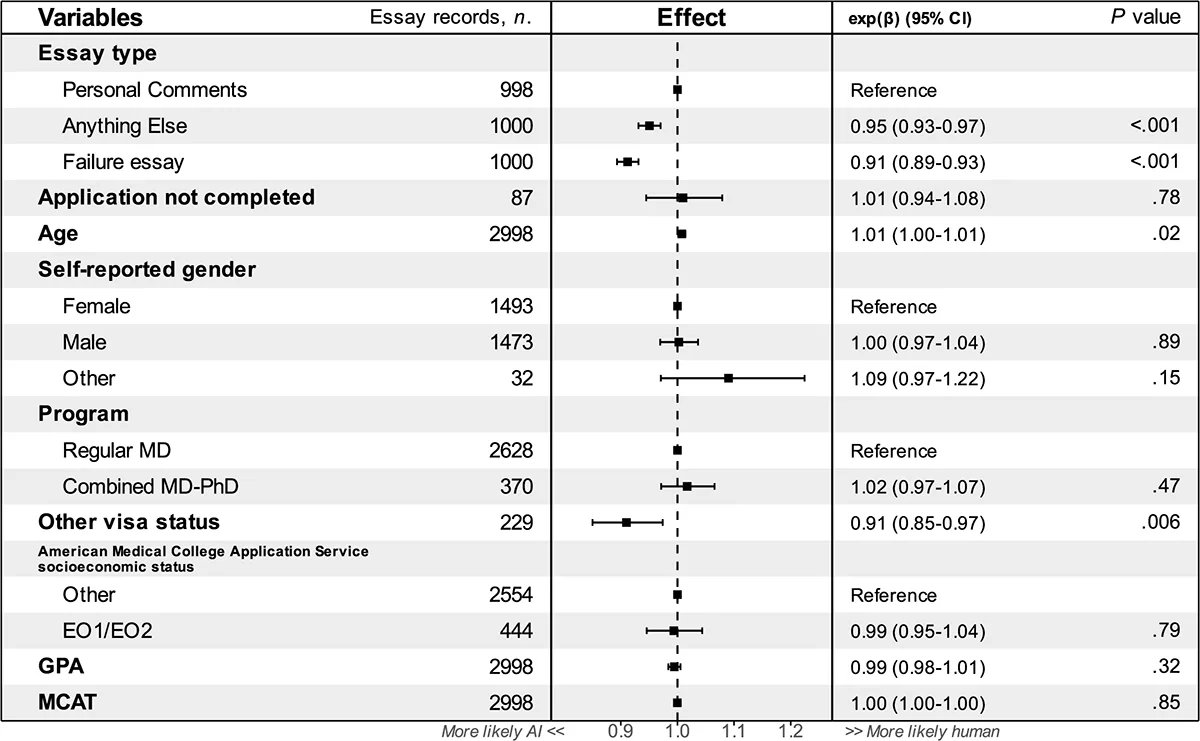
Applicant-clustered multivariable analysis of application-related factors associated with GPTZero-predicted P_human_ in the 2023 to 2024 application cycle. Estimates were obtained from a Gaussian identity-link generalized estimating equation with an independence working correlation and robust sandwich SEs clustered by coded American Medical College Application Service identifier. Black squares and horizontal lines show exp(β) and robust 95% CIs, respectively; these transformed identity-link coefficients are not odds ratios or risk ratios. The displayed *P* values are unadjusted, whereas the conclusions in the text are based on Benjamini-Hochberg–adjusted q values. Age was modeled per 1-year increase, Medical College Admission Test (MCAT) per 1-point increase, and grade point average (GPA) per 0.1-point increase.

The applicant-clustered multivariable GEE included 2998 essays from 1187 applicants. “Failure” essays (β=−.092, 95% CI −0.113 to −0.071; q<0.001) and “Anything Else” essays (β=−.051, 95% CI −0.071 to −0.03; q<0.001) had lower P_human_ values than “Personal Comments” essays (Figure 2). Older applicant age was associated with higher P_human_ (β=.0077 per year, 95% CI 0.0015-0.0139; q=0.043) and having other visa status was associated with lower P_human_ (β=−.094, 95% CI −0.162 to −0.027; q=0.024). Application completion, self-reported gender, program type, socioeconomic status, GPA, and MCAT score were not significant after false discovery rate correction.

In the completed application sensitivity analysis (2911 essays from 1105 applicants), both secondary essay types, age, and visa status remained significant after false discovery rate correction (age q=0.033; visa status q=0.021; Figure S2 in Multimedia Appendix 3). In the analysis restricted to applicants represented by all 3 selected essay types (2454 essays from 818 applicants), both secondary essay effects remained significant (both q<0.001), whereas the age and visa effect estimates remained in the same direction but were no longer significant after correction (age q=0.055; visa status q=0.133; Figure S3 in Multimedia Appendix 3). Complete clustered estimates are reported in the Supplementary Methods and Results sections in Multimedia Appendix 3.

We hypothesized that students who applied late might have been more likely to use AI to meet the deadline. Moreover, we hypothesized that applicants declaring only majors in STEM might be less comfortable with written expression and more likely to use AI. However, in exploratory analyses (data not shown), we found no correlation between P_human_ and the date of application submission (*r*=0.01) or the declaration of a STEM major (*P*=.50).

### Association Between Estimated AI Use and Admissions Outcomes

At the study school, application essays are used at every stage of review and could affect admissions decisions. We tested whether P_human_ was associated with interview invitation or medical school acceptance using adjusted applicant-level logistic regression models based on 1 “Personal Comments” essay per applicant (Table 3). Interview invitation was associated with male gender (odds ratio [OR] 0.51, 95% CI 0.37-0.72; q<0.001), EO1 and/or EO2 socioeconomic status (OR 3.31, 95% CI 2.08-5.29; q<0.001), MCAT score (OR 1.19 per point, 95% CI 1.14-1.24; q<0.001), and GPA (OR 1.81 per 0.1-point increase; q<0.001). Acceptance was associated with EO1 and/or EO2 socioeconomic status (OR 3.36; q<0.001), age (OR 1.17 per year; q=0.019), MCAT score (OR 1.18 per point; q<0.001), and GPA (OR 1.61 per 0.1-point increase, 95% CI 1.24-2.19 ; q=0.002). P_human_ was not associated with interview invitation (OR 1.61, 95% CI 0.87-3.02; q=0.2) or acceptance (OR 1.14, 95% CI 0.46-2.96; q=0.8).

**Table 3:** Adjusted applicant-level logistic regression analyses of factors associated with admissions outcomes in applicants with complete data from the 2023 to 2024 admissions cycle (N=815).

Characteristics			Interviewed applicants (n=142, 17%)							Accepted applicants (n=79, 10%)				
			OR^a^ (95% CI)		*P* value		q value^b^		OR (95% CI)			*P* value		q value
P_human_^c^			1.61 (0.87-3.02)		.13		0.19		1.14 (0.46-2.96)			.80		0.81
Age (years)			1.09 (1.00-1.18)		.04		0.061		1.17 (1.03-1.3)			.01		*0.019*
Self-reported gender														
	Female	—^d^		—		—		—			—		—	
	Male	0.51 (0.37-0.72)		<.001		*<0.001*		0.59 (0.35-0.97)			.04		0.064	
Program														
	Regular MD	—		—		—		—			—		—	
	Combined MD-PhD	0.76 (0.45-1.25)		.30		0.30		1.79 (0.95-3.27)			.06		0.084	
Visa status														
	US citizen	—		—		—		—			—		—	
	Other visa status	1.95 (0.99-3.75)		.049		0.066		1.4 (0.5-3.41)			.50		0.58	
American Medical College Application Service socioeconomic status														
	Other	—		—		—		—			—		—	
	EO1 and/or EO2	3.31 (2.08-5.29)		<.001		*<0.001*		3.36 (1.8-6.15)			<.001		*<0.001*	
Medical College Admission Test			1.19 (1.14-1.24)		<.001		*<0.001*		1.18 (1.11-1.26)			<.001		*<0.001*
Grade point average			1.81 (1.50-2.22)		<.001		*<0.001*		1.61 (1.24-2.19)			<.001		*0.002*

^a^OR: odds ratio. For age, the OR gives the change associated with a 1-year increase; for the Medical College Admission Test, a 1-point increase in the 3-digit score; and for grade point average, a 0.1-point increase in undergraduate grade point average.

^b^False discovery rate–adjusted *P* values for multiple testing. Italics indicate *q*≤0.05.

^c^P_human_ refers to GPTZero’s determination for the “Personal Comments” essay.

^d^Not applicable.

## Discussion

### Principal Findings

We report our analysis of a natural experiment, comparing a cohort of medical school applicants who applied in 2021 to 2022, before LLM-driven chatbots were widely available, with the cohort who applied in 2023 to 2024, after the release of ChatGPT and GPT-3/4, when chatbots had become easily accessible, free, and frequently discussed in mainstream media and academic circles.

In the baseline year, 2.7% of essays had P_human_<0.5 and therefore showed a style signal suggesting that AI was more likely than not to have been used in some way during preparation. Although some essays might have used earlier generative AI tools, this finding more likely reflects the population-wide lower limit of the detector due to false-positive determinations. Our major finding was a 9.6-percentage-point higher proportion of essays below this threshold in the 2023 to 2024 cycle. In the applicant-clustered multivariable GEE, lower P_human_ was independently associated with secondary essay type, younger age, and other visa status; GPA and application completion were not associated after false discovery rate correction. There was no evidence of an association with socioeconomic status or undergraduate major, and we did not find evidence that late applicants were more likely to have lower P_human_. P_human_ was not significantly associated with interview invitations or acceptance decisions.

### Strengths

Strengths of this paper include the large dataset, the use of multiple time points (bracketing the introduction of widely available generative AI), and the use of control inputs. Applicants to the study school represented approximately 10% of the 49,570 individuals who applied to US medical schools through AMCAS in 2023 to 2024. The 2021 to 2022 group had, at most, rare and sporadic access to AI and therefore provides a benchmark against which the 2023 to 2024 group can be compared. Additionally, the highly confident positive predictions for the positive controls and negative predictions for the negative controls support the validity of the methods.

An additional strength was the explicit modeling of within-applicant correlation among repeated essays. The similar essay-type estimates in the full, completed application and all-3-essay clustered models support the robustness of the primary essay-type findings to variation in an applicant’s propensity to use generative AI for any essay, as well as to the unequal numbers of selected essays per applicant.

### Limitations

Limitations of the paper include its single-site nature and the fact that the applicants to the study school have, on average, higher academic achievement than the AMCAS applicant pool as a whole and may otherwise be nonrepresentative. In our analysis of AI use in relation to application outcomes, we had information only on the admissions actions at the study school; some applicants rejected by the study school were undoubtedly accepted at other schools.

For practical reasons, we used only one AI detector, although several are available. GPTZero was chosen due to its wide adoption and the availability of an application programming interface. Moreover, the accuracy of a similar algorithm was 91% in a study that compared human-authored personal statements to known synthetic ones [13], and to be at least 96% in a larger benchmarking study of diverse writing samples [7]. Subsequently, new tools have been released with incrementally better performance and could be applied in future studies. There may also be other approaches, such as applicant surveys, to learn about AI use. These approaches could provide a direct assessment of AI use rather than an inferred assessment but would be limited by the honesty of the respondents.

A methodological limitation of this project is that the distinction between human-written and AI-written text is excessively dualistic. Although some applicants may blithely copy AI output directly into their application materials, it seems more plausible that they will adapt the AI text to their own situation, thus moving some or all sentences away from being purely AI generated. Such edited text could pass for human-written although AI would have played a part in producing it. For the present study, we assumed that at least some AI-derived text remains detectable after human editing. We used controls to understand how GPTZero scores known human and known AI essays, but we did not study its behavior in less dualistic scenarios (eg, asking AI to tweak known human essays, using humans to tweak AI essays, or manually blending human and AI inputs).

An important additional caveat is that AI detection is known to be imperfect [14,15]. The sensitivity and specificity of AI detectors can be tuned and generally are set so that the specificity is high at the expense of sensitivity. For example, in one small-scale study involving 50 writing samples, GPTZero was benchmarked at a sensitivity of 65% and a specificity of 90%, against an overall accuracy of 80% [14]. A larger study found a false-negative rate of between 0.2% and 3%, depending on the model used to generate the input text, and a false-positive rate of less than 1% [7]. The tradeoff is biased toward higher specificity because the consequences of a false-positive error (false detection of AI use, potentially leading to wrongful accusations of academic malfeasance or policy noncompliance) are less palatable than the consequences of a false-negative error. The result, however, is that some AI-generated text will be classified as human generated. Moreover, AI detectors may be more effective in identifying earlier and less advanced iterations of AI chatbots [9], whereas newer models generate more humanlike output and, in some cases have been specifically designed to evade detection. Prompt engineering can be used to direct chatbots to write more like humans, and the resulting text is less readily detected [16,17]. As some chatbots are marketed on a freemium model (free tier or paid tier), students with greater financial resources may have access to more sophisticated versions. Anecdotally, some types of inputs, such as lists, may be erroneously flagged as AI generated. This failure mode could lead to inaccurate detection in medical school application materials, which sometimes include lists of students’ activities or publications. Together, these factors could lead to unjust outcomes if AI detection were deployed with real-world consequences (eg, disqualifying applicants for detected use of AI).

AI detectors may misclassify the authentic work of nonnative English writers as AI generated [16] due to their more restricted vocabulary and syntax. Indeed, we found that applicants requiring a visa to study in the United States had a lower probability of human-generated text, which may reflect higher AI use but could also reflect this failure mode of AI detection. English-language learners may also be more likely to use AI to proofread and correct authentic human-written essays. We do not know how AI-assisted “proofreading” affects P_human_, compared with outright composition of writing samples by AI, for which we have benchmarked P_human_. Because of these factors, reliance on AI detection could unintentionally penalize English-language learners.

As a final caveat, the importance placed on application essays may vary from one school to another. The impact of AI use on admissions outcomes would vary accordingly. There may be variation between schools in the propensity of their respective applicant pools to use AI. The structure of the application (including the number and length of essays and the time permitted to complete the secondary application) could be an underlying factor. Our findings suggest that lengthy secondary applications are more likely to be completed with AI assistance. Students applying to more or less selective schools might be more or less likely to use AI. The magnitude and even the direction of this effect are not known.

### Comparison With Prior Work

To the best of our knowledge, this is the first real-world study of generative AI use by medical school applicants. A recent study studied a smaller set of 32 essays in the style of medical school applicant essays, written explicitly for the purposes of the study using varying degrees of AI assistance. The authors found no association between AI use and raters’ subjective assessment of these essays [18], suggesting that applicants gain little advantage through AI use, similar to our findings.

Another study by Vaccaro et al [13] compared a baseline set of essays written before the advent of generative chatbots to a second set of essays generated using AI. Human readers were unable to distinguish between human-written and AI-written essays and assigned marginally higher scores to the AI-written essays. The study by Vaccaro et al [13] provides validity evidence for our use of linguistic-pattern–based AI detection. The authors concluded that AI use threatens the use of personal statements in admissions, whereas our assessment is more sanguine given the lack of an impact on admissions outcomes.

There is evidence that writers who are not medical school applicants are using generative AI as well. The editors of the journal *Organization Science* used a methodology similar to ours, but with a different AI detector, and found evidence of a marked increase in AI-written submissions to their journal, accompanied by a decrease in quality, after the public launch of ChatGPT in 2022 [19]. Although we found that AI use conferred no admissions advantage (or disadvantage), the authors found that AI-written papers were more likely to be rejected. The authors concluded that AI-written submissions are placing stress on the peer review ecosystem. It remains to be seen what stressors AI will place on medical school admissions.

### Conclusions

Our results suggest that approximately 10% of medical school application essays were written with the help of generative AI in the first application cycle after these tools became widely available. Based on data from a single medical school, there appeared to be no correlation between AI use and admissions outcomes.

Given the difficulty of definitively identifying AI use in applicant essays and the lack of clarity regarding the appropriate response, we recommend continuing to study such data only in aggregate and on an informational basis. If there were evidence to suggest widespread AI use in answering a specific item, for example, the appropriate institutional response might be to design a new item that is less amenable to AI rather than to penalize applicants who appear to be providing AI-generated answers. Similarly, we do not recommend that schools adopt software that would automatically flag AI use at the level of an individual applicant, although such software is likely to become available in the future. Playing a cat-and-mouse game around AI can only induce cynicism and erode overall trust in the application process, with the potential for unjust outcomes or the exacerbation of disparities.

It is unlikely that AI use can or should be entirely eliminated from medical school applications. Indeed, the 2025 AMCAS application contains a revised certification statement in which the applicant must agree that although AI use is permitted, the final product must be “a true reflection of [their] own work and represents [their] experiences” [20]. Applicants’ reliance on AI could be a symptom of the heavy cognitive burden associated with preparing a complete medical school application. As secondary applications showed more evidence of AI use than the common AMCAS application, schools should consider whether there is a benefit in including additional writing samples in their school-specific secondary applications. These one-off tasks are numerous and are completed on a shorter timeline than the AMCAS essay, which could put applicants under pressure to take shortcuts. In turn, prehealth advisers should inform applicants that using AI deprives them of the opportunity to tell their own story and highlight the unique contribution they will make to medicine.

## Data Availability

Due to the nature of the study, the data are not directly available but may be made available upon reasonable request.

## Appendix

Multimedia Appendix 1 Classification of undergraduate major subjects as science, technology, engineering, and mathematics (STEM) vs non-STEM.

Multimedia Appendix 2 Positive-control essays generated using AI chatbots.

Multimedia Appendix 3 Supplemental methods and results.

## Abbreviations

AMCAS: American Medical College Application Service
AUROC: area under the receiver operating characteristic
GEE: generalized estimating equation
GPA: grade point average
LLM: large language model
MCAT: Medical College Admission Test
OR: odds ratio
STEM: science, technology, engineering, and mathematics
