# Supplementary material for: Estimating the Prevalence of Generative AI Use in Medical School Application Essays: Cross-Sectional Study
Source: JMIR Med Educ. 2026 Sep 3;12:e96673. doi: 10.2196/96673 (PMC13586704; doi:10.2196/96673)
Supplement: Supplementary file 3 [file mededu_v12i1e96673_app3.docx]

Supplementary Methods and Results

Clustering Diagnostics and Sensitivity Analyses

# Supplementary Methods

## Repeated-observation diagnostics and application-cycle sensitivity analysis

The primary sampling procedure, outcome definition, and applicant-clustered models are described in the main manuscript. For these supplemental analyses, we summarized the number of selected essay types contributed by each applicant-cycle and identified coded AMCAS identifiers occurring in both application cycles.

To quantify the effect of within-applicant dependence on the application-cycle comparison, we compared an unclustered Gaussian identity-link model with an otherwise identical generalized estimating equation (GEE) using an independence working correlation and robust sandwich standard errors clustered by coded AMCAS identifier. Both models included application cycle, essay type, and their interaction. Overall cycle contrasts were standardized with equal weight across the 3 essay types, and essay-specific contrasts used Holm-adjusted P values. We additionally fit an exchangeable-working-correlation GEE and estimated the applicant-level intraclass correlation coefficient (ICC) from a Gaussian random-intercept model.

## Application-factor sensitivity analyses

The main manuscript reports the primary applicant-clustered multivariable GEE. Supplemental Table 5 compares that model with a corresponding unclustered linear model fit to the same complete-case sample solely to show how accounting for clustering affected statistical uncertainty. With an identity link and independence working correlation, the models yield identical coefficients but different standard errors. Singleton applicant clusters were retained, and Benjamini-Hochberg correction was applied separately within each model.

Two restricted-sample GEEs were done to evaluate sensitivity to application and essay availability. One included only applicants classified as having a completed application, defined as a nonmissing application-submission date. The other included only applicants represented in the analytic sample by exactly 1 Personal Comments, 1 Failure, and 1 Anything Else essay, thereby eliminating variation in cluster size. Both used the primary covariate set except application completion, which was fixed by design in the completed-application subset and nearly invariant in the all-3-essay subset, where only 1 of 818 applicants was classified as incomplete. False discovery rate correction was applied across the 10 nonintercept coefficients in each restricted model. All tests were 2-sided. Analyses used R version 4.5.1 and geepack version 1.3.13.

# Supplementary Results

## Applicant overlap

Approximately 31% of applicant-cycles contributed fewer than all 3 selected essay types: 187 applicants contributed 1 essay and 178 contributed 2 in 2021–2022; the corresponding counts were 196 and 175 in 2023–2024 (**Supplemental Table 3**). The 2,373 applicant-cycle observations represented 2,364 unique coded identifiers because 9 identifiers occurred in both cycles.

**Supplemental Table 3. Number of selected essays contributed per applicant-cycle.**

| Measure | 2021–2022 | 2023–2024 |
| --- | --- | --- |
| Essay records, n | 3,000 | 3,000 |
| Applicants, n | 1,184 | 1,189 |
| Essays per applicant, mean (SD) | 2.53 (0.75) | 2.52 (0.76) |
| Essays per applicant, median (IQR) | 3 (2–3) | 3 (2–3) |
| Applicants with 1 selected essay, n (%) | 187 (15.8%) | 196 (16.5%) |
| Applicants with 2 selected essays, n (%) | 178 (15.0%) | 175 (14.7%) |
| Applicants with 3 selected essays, n (%) | 819 (69.2%) | 818 (68.8%) |

## Effect of clustering on the application-cycle comparison

Relative to the unclustered model, applicant-clustered inference increased the standard error of the standardized application-cycle contrast from 0.0072 to 0.0099, a 36.7% increase, while leaving its point estimate unchanged (**Supplemental Table 4**). The exchangeable GEE estimated a within-applicant working correlation of 0.497 and produced a similar cycle difference of −0.140 (95% CI −0.158 to −0.121; P<.001). The random-intercept ICC was 0.483.

The clustered application cycle-by-essay type interaction was significant (χ²₂=20.95; P<.001). *P*_human_ was lower in 2023–2024 for Personal Comments (difference −0.115, 95% CI −0.136 to −0.095), Anything Else essays (−0.142, 95% CI −0.168 to −0.117), and Failure essays (−0.176, 95% CI −0.203 to −0.149); all Holm-adjusted P values were <.001.

**Supplemental Table 4. Impact of applicant clustering on the overall application-cycle contrast.**

| Model | Difference, 2023–2024 minus 2021–2022 | SE | 95% CI | P value | Interaction P value |
| --- | --- | --- | --- | --- | --- |
| Unclustered Gaussian linear model | −0.145 | 0.0072 | −0.159 to −0.130 | <.001 | .003 |
| Applicant-clustered robust GEE, independence working correlation | −0.145 | 0.0099 | −0.164 to −0.125 | <.001 | <.001 |
| Applicant-clustered GEE, exchangeable working correlation | −0.140 | 0.0096 | −0.158 to −0.121 | <.001 | <.001 |

*Note.* Overall contrasts were standardized with equal weight across the 3 essay types. The independence-working-correlation GEE has the same point estimate as the unclustered model and isolates the impact of clustered robust inference. The exchangeable GEE additionally models a common within-applicant correlation.

## Effect of clustering on application-factor estimates

As expected, clustering did not change the multivariable coefficients but altered their precision (Supplemental Table 5). Robust standard errors were approximately 28–29% smaller for the within-applicant essay-type contrasts and generally larger for applicant-level characteristics; for example, they increased by approximately 52% for visa status and 58% for GPA. No predictor changed false discovery rate significance classification. Thus, the unclustered comparison affected the estimated uncertainty but not the substantive interpretation of the primary clustered model reported in the main manuscript (**Supplemental Figure 1**).

**Supplemental Table 5. Multivariable application-factor estimates before and after accounting for applicant clustering.**

| Predictor | Unclustered β (95% CI) | Unclustered q value | Applicant-clustered β (95% CI) | Clustered q value |
| --- | --- | --- | --- | --- |
| Failure essay vs Personal Comments | −0.092 (−0.121 to −0.063) | <.001 | −0.092 (−0.113 to −0.071) | <.001 |
| Anything Else vs Personal Comments | −0.051 (−0.080 to −0.022) | .002 | −0.051 (−0.071 to −0.030) | <.001 |
| Application not completed | 0.010 (−0.064 to 0.083) | .848 | 0.010 (−0.057 to 0.076) | .892 |
| Age, per year | 0.0077 (0.0025 to 0.0129) | .010 | 0.0077 (0.0015 to 0.0139) | .043 |
| Male vs female | 0.002 (−0.021 to 0.026) | .848 | 0.002 (−0.031 to 0.036) | .892 |
| Other gender vs female | 0.086 (−0.028 to 0.201) | .256 | 0.086 (−0.030 to 0.202) | .320 |
| Combined MD/PhD vs regular MD | 0.017 (−0.019 to 0.053) | .546 | 0.017 (−0.029 to 0.063) | .738 |
| Other visa status vs U.S. citizen | −0.094 (−0.139 to −0.050) | <.001 | −0.094 (−0.162 to −0.027) | .024 |
| EO1/EO2 vs other socioeconomic status | −0.007 (−0.040 to 0.027) | .848 | −0.007 (−0.056 to 0.043) | .892 |
| GPA, per 0.1 point | −0.0055 (−0.0123 to 0.0013) | .250 | −0.0055 (−0.0162 to 0.0052) | .578 |
| MCAT, per point | −0.0003 (−0.0023 to 0.0017) | .848 | −0.0003 (−0.0034 to 0.0028) | .892 |

*Note.* β coefficients are reported on the *P*_human_ scale. The applicant-clustered model is a Gaussian identity-link GEE with an independence working correlation and robust sandwich standard errors clustered by coded AMCAS identifier. *q* values use the Benjamini-Hochberg false discovery rate correction within each model.

**Supplemental Figure 1.** Comparison of coefficients and 95% confidence intervals from the primary applicant-clustered multivariable GEE and the corresponding unclustered comparison model.

## Restricted-sample analyses

Restricting the model to applicants with completed applications produced estimates closely aligned with the primary analysis, with no additional predictors reaching false discovery-rate significance (**Supplemental Figure 2**). In the balanced all-3-essay subset, the secondary essay-type contrasts were slightly larger in magnitude than in the primary model. Age and visa-status estimates remained in the same directions, but their greater uncertainty and multiple-testing adjustment moved them above the false discovery rate threshold; no other predictors became significant (**Supplemental Figure 3**). These analyses support the robustness of the essay-type findings while showing that the weaker age and visa-status findings were sensitive to the smaller restricted sample.

**Supplemental Figure 2.** Complete estimates from the applicant-clustered multivariable analysis restricted to applicants with completed applications. Black squares and horizontal lines show exp(β) and robust 95% confidence intervals, respectively; these transformed identity-link coefficients are not odds ratios or risk ratios. Plotted P values are unadjusted, whereas conclusions use Benjamini-Hochberg-adjusted q values. N values are essay records.

**Supplemental Figure 3.** Complete estimates from the applicant-clustered multivariable analysis restricted to applicants represented in the analytic sample by all 3 selected essay types. Plotting conventions are as described for Supplemental Figure 2; each applicant contributed 1 essay record of each type.
